# Radiologist Perceptions of an AI Tool for Intracranial Hemorrhage Detection in Teleradiology: Cross-Sectional Survey Study

**DOI:** 10.2196/92145

**Published:** 2026-06-02

**Authors:** Andrew J Del Gaizo, Jackson K Del Gaizo, Troy A Shahoumian

**Affiliations:** 1 Moffitt Cancer Center Tampa, FL United States; 2 Durham Academy Durham, NC United States; 3 Population Health Informatics, Digital Health Office Veterans Health Administration Washington DC, DC United States

**Keywords:** artificial intelligence, AI, intracranial hemorrhage, computed tomography, teleradiology, workflow, false positives, trust in automation, alert fatigue, human factors, decision support

## Abstract

**Background:**

Artificial intelligence (AI) detection tools for intracranial hemorrhage (ICH) are increasingly integrated into radiology workflows. In real-world practice, perceived utility depends not only on diagnostic performance but also on workflow fit, false positive burden, and how clinicians interpret and act on AI outputs.

**Objective:**

This study aimed to characterize radiologists’ perceptions of a Food and Drug Administration (FDA)–cleared ICH AI detection tool in a national teleradiology network, including perceived reliability, false positive burden, workflow impact, medicolegal concerns, and self-reported behaviors during routine use.

**Methods:**

We conducted an anonymous cross-sectional survey of radiologists in a national teleradiology practice who had access to an FDA-cleared ICH AI overlay during noncontrast head computed tomography interpretation. Survey items used a 5-point Likert scale. Results are summarized as agreement proportions (“agree” or “strongly agree”) with 95% CIs. We compared neuroradiologists with non-neuroradiologists using Fisher exact tests. One primary end point was prespecified: agreement that time spent reviewing examinations with false positive AI alerts outweighed the benefits. Remaining subgroup comparisons were treated as exploratory, with false discovery rate control using the Benjamini-Hochberg procedure.

**Results:**

A total of 65 radiologists responded, including 23 (35.4%) neuroradiologists and 42 (64.6%) non-neuroradiologists. Only 18.5% (12/65; 95% CI 10.9%-29.6%) agreed that false-positive alerts were infrequent enough to be acceptable. Agreement that the AI correctly identified most ICH cases was 32.3% (21/65; 95% CI 22.2%-44.4%), and agreement that the AI rarely missed clinically important hemorrhages was 43.1% (28/65; 95% CI 31.8%-55.2%). Trust in AI output was conditional: 50.8% (33/65; 95% CI 38.9%-62.5%) reported trusting the AI when it agreed with their interpretation, whereas 3.1% (2/65; 95% CI 0.8%-10.5%) reported trusting it when it conflicted with their interpretation. Only 10.8% (7/65; 95% CI 5.3%-20.6%) reported reduced overall interpretation time, whereas 33.8% (22/65; 95% CI 23.5%-46.0%) agreed that time spent reviewing false-positive alerts outweighed the benefits. Self-reported reduced scrutiny after an AI-negative result was uncommon (4/65, 6.2%; 95% CI 2.4%-14.8%). In subgroup analysis, neuroradiologists more often endorsed the primary end point than non-neuroradiologists (12/23, 52.2% vs 10/42, 23.8%; unadjusted *P*=.03), but no exploratory subgroup differences remained statistically significant after false discovery rate correction. Free-text responses emphasized artifact- and calcification-driven false positives, delayed or inconsistent AI availability, consultation burden, and medicolegal concerns.

**Conclusions:**

In this national teleradiology setting, radiologists reported substantial false positive burden, limited perceived time savings, and strongly conditional trust in an FDA-cleared ICH AI detection tool. Self-reported reduced scrutiny after negative AI outputs was uncommon but present in a minority of cases. These findings support the importance of specificity, interpretability, latency, and workflow-aware implementation when deploying radiology AI tools in practice.

## Introduction

Intracranial hemorrhage (ICH) on noncontrast head computed tomography (CT) is a high-stakes diagnosis in emergency and inpatient care requiring timely identification and communication. Artificial intelligence (AI) systems intended to detect ICH are increasingly incorporated into radiology workflows, including as diagnostic assistive tools during radiologist interpretation and, in some implementations, as tools to support prioritization of urgent cases [1-4]. However, the real-world value of these systems depends not only on discriminative performance but also on how they interact with clinical workflow, how often they generate disruptive false-positive alerts, and how clinicians interpret their outputs in routine practice [2-5].

Post-deployment evaluation is increasingly recognized as essential for clinical AI. DECIDE-AI (Developmental and Exploratory Clinical Investigations of Decision Support Systems Driven by Artificial Intelligence) and related implementation literature emphasize that early live clinical evaluation should address workflow integration, human factors, and unintended burdens rather than focusing on accuracy alone [5]. Real-world studies of radiology AI have similarly shown that local prevalence, operational context, and implementation strategy can substantially shape perceived utility and clinician acceptance [2,3,6]. For example, deployment experience with an AI algorithm in clinical practice has highlighted how false discovery burden can create an “accuracy paradox” in which an apparently high-performing system is experienced as inaccurate because positive alerts are frequently false in a low-prevalence setting [6].

Human factors theory suggests that false alarms, workflow interruptions, and opaque outputs can alter clinician reliance on automation [7-10]. In health care more broadly, high alert burden and repeated alerts can contribute to alert fatigue and reduced responsiveness to clinical decision support alerts [8]. In radiology, incorrect AI outputs may affect reader confidence and performance, particularly when the AI appears authoritative or visually salient [9]. At the same time, survey data alone cannot establish whether AI changes diagnostic behavior; they are better suited to characterizing clinician perceptions, attitudes, and self-reported experiences.

We previously evaluated a Food and Drug Administration (FDA)–cleared ICH AI tool in a national teleradiology network and reported operational observations, including false positives and radiologist-reported effects on interpretation time [1]. The study reported in this paper extends that work by focusing on radiologists’ perceptions during routine exposure to the tool. Specifically, we sought to characterize perceived reliability, false positive burden, workflow impact, medicolegal concerns, and self-reported behaviors during AI-assisted interpretation and explore whether these perceptions differed by neuroradiology subspecialty training.

## Methods

### Study Design

This was an anonymous cross-sectional survey study conducted within a national teleradiology practice. This study was designed as a real-world implementation evaluation aligned with early-stage clinical AI assessment principles, with emphasis on workflow context and user experience [5].

### Ethical Considerations

This evaluation met the US Department of Veterans Affairs criteria for quality assessment and nonresearch and received a determination of nonresearch from the Stanford University Institutional Review Board (protocol 73642). Participation was voluntary and anonymous.

### Participants

Eligible participants were practicing radiologists within the teleradiology group who had access to the ICH AI output during routine noncontrast head CT interpretation. Respondents self-reported whether they had neuroradiology fellowship training or primarily practiced neuroradiology, and responses were stratified as neuroradiologists vs non-neuroradiologists.

### AI Tool and Workflow Context

The evaluated system was an FDA-cleared and commercially available deep learning algorithm for ICH detection (CINA version 1.0; Avicenna.ai) integrated into the reading workflow for noncontrast head CT. The tool was intended to assist radiologists in identifying suspected ICH during image interpretation and, depending on workflow configuration, could also support prioritization. In practice, radiologists could view AI-generated overlays during interpretation, and the system functioned as an adjunctive diagnostic support tool within routine workflow. The tool did not replace radiologist interpretation, and final clinical interpretation remained the responsibility of the reading radiologist. Because intended use and actual workflow exposure are both relevant to interpretation of user perceptions, we frame these findings as perceptions arising during routine use of the tool in this implementation context.

### Survey Instrument

The survey included 15 Likert items spanning the following domains: perceived reliability, false positive burden, workflow integration, time impact, medicolegal concerns, adoption preferences, and self-reported behaviors during AI use. Responses were captured on a 5-point scale (“strongly agree,” “agree,” “neutral,” “disagree,” and “strongly disagree”). Items were developed for face validity based on prior operational experience and relevant human factors concepts, but the instrument was not a formally validated psychometric measure. Accordingly, results were interpreted on the face of each item as descriptive measures of respondent perceptions.

### Prespecified End Point and Statistical Analysis

To limit overinterpretation from multiple comparisons, one primary end point was prespecified: agreement with the following statement: “The extra time spent reviewing AI false-positive studies outweighs the benefits.” This end point was selected because false positive burden was considered a central determinant of usability and adoption in a high-volume teleradiology environment.

All other item-level subgroup comparisons were considered exploratory. Responses were summarized as agreement proportions, defined as the percentage selecting “agree” or “strongly agree.” We report proportions with 2-sided 95% CIs using Wilson intervals. For subgroup comparisons, we used Fisher exact tests on dichotomized responses (“agree” or “strongly agree” vs all other responses). Because responses were condensed from a 5-point scale to a binary end point for interpretability and consistency with implementation-focused survey reporting, some resolution was necessarily lost. Therefore, we interpret subgroup analyses as descriptive and hypothesis generating. False discovery rate control for exploratory subgroup comparisons was applied using the Benjamini-Hochberg procedure. Analyses were performed in Python (version 3.13.5; Python Software Foundation) using exact tests and Wilson interval estimation with *SciPy* (version 1.17.0) and *statsmodels* (version 0.14.6).

### Qualitative Analysis

Optional free-text comments were reviewed qualitatively to identify recurring implementation themes. Comments could map to more than one theme. Representative excerpts were selected to illustrate the most common concerns raised by respondents.

## Results

### Respondent Characteristics

A total of 65 radiologists completed the survey, including 23 (35.4%) neuroradiologists and 42 (64.6%) non-neuroradiologists. Full quantitative results across all survey items are provided in Table 1.

**Table 1:** Agreement proportions across survey items (“agree” or “strongly agree”) overall and stratified by neuroradiology training. The prespecified primary end point is item 7 (N=65)^a^.

Item number	Question	Total, n (%; 95% CI)	Neuroradiologists (n=23), n (%)	Non-neuroradiologists (n=42), n (%)	*P* value	*q* value
1	The AI^b^ tool correctly identifies most cases of intracranial hemorrhage.	21 (32.3; 22.2-44.4)	8 (34.8)	13 (31.0)	.79	.92
2	False-positive alerts from the AI are infrequent enough to be acceptable.	12 (18.5; 10.9-29.6)	4 (17.4)	8 (19.0)	>.99	>.99
3	The AI rarely misses clinically important hemorrhages.	28 (43.1; 31.8-55.2)	10 (43.5)	18 (42.9)	>.99	>.99
4	I trust the AI output when it agrees with my own interpretation.	33 (50.8; 38.9-62.5)	11 (47.8)	22 (52.4)	.80	.92
5	I trust the AI output when it conflicts with my initial impression.	2 (3.1; 0.8-10.5)	0 (0)	2 (4.8)	.54	.90
6	Using the AI has reduced my overall interpretation time per head CT^c^.	7 (10.8; 5.3-20.6)	1 (4.3)	6 (14.3)	.41	.90
7	The extra time spent reviewing AI false-positive studies outweighs the benefits.	22 (33.8; 23.5-46.0)	12 (52.2)	10 (23.8)	.03	—^d^
8	If the AI labels a study as negative, I tend to scrutinize it less thoroughly than usual.	4 (6.2; 2.4-14.8)	0 (0)	4 (9.5)	.29	.90
9	The AI reduces the risk that I will overlook a subtle hemorrhage on a busy shift.	23 (35.4; 24.9-47.5)	7 (30.4)	16 (38.1)	.60	.90
10	Frequent false positive alerts make me second-guess my decisions.	20 (30.8; 20.9-42.8)	4 (17.4)	16 (38.1)	.10	.75
11	Overall, the AI has improved the quality of care our patients receive.	13 (20; 12.1-31.3)	4 (17.4)	9 (21.4)	.76	.92
12	I feel legally vulnerable if I override an AI recommendation.	21 (32.3; 22.2-44.4)	6 (26.1)	15 (35.7)	.58	.90
13	The AI integrates smoothly into our PACS^e^/reporting workflow.	32 (49.2; 37.5-61.1)	13 (56.5)	19 (45.2)	.44	.90
14	I would recommend continued use of this AI tool in our practice.	19 (29.2; 19.6-41.2)	5 (21.7)	14 (33.3)	.40	.90
15	I would like additional feedback or training to use the AI tool optimally.	13 (20; 12.1-31.3)	3 (13.0)	10 (23.8)	.35	.90

^a^Between-group comparisons were conducted using Fisher exact tests. False discovery rate control across exploratory subgroup comparisons was applied using the Benjamini-Hochberg procedure; *q* values are reported for exploratory items.

^b^AI: artificial intelligence.

^c^CT: computed tomography.

^d^Not applicable. Item 7 was the prespecified primary end point; *q* values were not applied to this item.

^e^PACS: picture archiving and communication system.

### Perceived Reliability and False Positive Burden

Agreement that the AI correctly identified most cases of ICH was 32.3% (21/65; 95% CI 22.2%-44.4%). Agreement that the AI rarely missed clinically important hemorrhages was somewhat higher at 43.1% (28/65; 95% CI 31.8%-55.2%). In contrast, only 18.5% (12/65; 95% CI 10.9%-29.6%) agreed that false-positive alerts were infrequent enough to be acceptable, indicating that perceived false positive burden was a prominent limitation.

The prespecified primary end point was also consistent with this pattern. Overall, 33.8% (22/65) of respondents (95% CI 23.5%-46.0%) agreed that the extra time spent reviewing examinations with false positive AI alerts outweighed the benefits of the system. These radiologists endorsed the view that false positive workload offset the tool’s net value in routine use. Overall agreement across survey items is shown in Table 1.

### Trust in AI Output and Perceived Workflow Impact

Responses suggested strongly conditional trust in the AI output. Half (33/65, 50.8%) of respondents (95% CI 38.9%-62.5%) agreed that they trusted the AI when it agreed with their own interpretation. In contrast, only 3.1% (2/65; 95% CI 0.8%-10.5%) agreed that they trusted the AI when it conflicted with their initial impression. This asymmetry suggests that respondents were much more likely to experience the AI as confirmatory than as persuasive in discrepant cases.

Perceived workflow benefit was limited. Only 10.8% (7/65) of respondents (95% CI 5.3%-20.6%) agreed that AI use reduced their overall interpretation time per head CT. Similarly, 35.4% (23/65; 95% CI 24.9%-47.5%) agreed that the AI reduced the risk that they would overlook a subtle hemorrhage on a busy shift, indicating that perceived detection support was present for some respondents but did not translate into broad perception of time savings.

### Self-Reported Behaviors, Quality Perceptions, and Medicolegal Concerns

Self-reported reduced scrutiny after a negative AI result was uncommon. Only 6.2% (4/65) of the respondents (95% CI 2.4%-14.8%) agreed with the statement that, if the AI labeled a result as negative, they tended to scrutinize it less thoroughly than usual. Because this was a self-reported perception and not a direct behavioral measurement, we interpreted it narrowly as a descriptive response to the survey item.

Frequent false-positive alerts were also associated with self-reported second-guessing. A total of 30.8% (20/65) of the respondents (95% CI 20.9%-42.8%) agreed that frequent false-positive alerts made them second-guess their decisions. Only 20% (13/65; 95% CI 12.1%-31.3%) agreed that the AI improved the quality of care that patients received. Agreement that the AI integrated smoothly into the picture archiving and communication system or reporting workflow was 49.2% (32/65; 95% CI 37.5%-61.1%), suggesting mixed perceptions of integration. Recommendation for continued use of the tool was endorsed by 29.2% (19/65; 95% CI 19.6%-41.2%). Concern about legal vulnerability when overriding AI output was reported by 32.3% (21/65; 95% CI 22.2%-44.4%). Additional feedback or training to use the tool optimally was desired by 20% (13/65; 95% CI 12.1%-31.3%).

### Subgroup Findings by Neuroradiology Training

**Figure 1:**
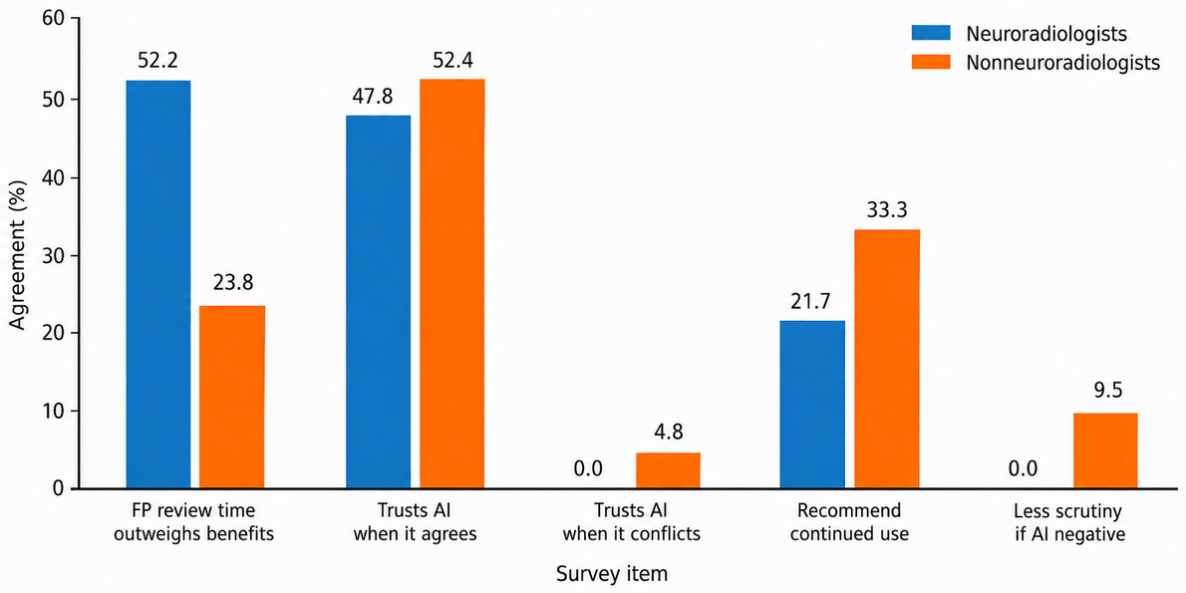
Descriptive comparison of agreement by subspecialty training for selected survey items. Bars show the percentage of respondents selecting “agree” or “strongly agree” among neuroradiologists and non-neuroradiologists for selected items related to false positive (FP) burden, trust in artificial intelligence (AI) output, recommendation for continued use, and reported less thorough than usual scrutiny of AI-negative results. These subgroup comparisons are descriptive; no exploratory subgroup differences remained statistically significant after false discovery rate correction.

Neuroradiologists more frequently endorsed the primary end point than non-neuroradiologists (12/23, 52.2% vs 10/42, 23.8%; unadjusted *P*=.03). For exploratory items, patterns were broadly similar between groups. Agreement with trusting the AI when it agreed with the reader’s interpretation was 47.8% (11/23) among neuroradiologists and 52.4% (22/42) among non-neuroradiologists. Trust in conflicting AI output remained rare in both groups (0% vs 2/42, 4.8%). Self-reported reduced scrutiny after an AI-negative result was reported by 0% of neuroradiologists and 9.5% (4/42) of non-neuroradiologists. Recommendation for continued use was reported by 21.7% (5/23) of neuroradiologists and 33.3% (14/42) of non-neuroradiologists. After false discovery rate correction, no exploratory subgroup differences remained statistically significant. Descriptive subgroup comparisons for selected survey items are shown in Figure 1.

### Qualitative Themes From Free-Text Comments

Free-text responses reinforced the quantitative findings and highlighted recurring implementation concerns.

#### Theme 1: Artifact- and Calcification-Driven False Positives Dominated the User Experience

Many respondents attributed false-positive alerts to parafalcine calcification, skull-adjacent artifacts, motion, or streak artifacts and described these alerts as disruptive or time-consuming.

#### Theme 2: Perceived Utility Was Limited Unless the Tool Meaningfully Changed Triage

Several respondents indicated that the system primarily identified obvious hemorrhages and offered limited incremental value for experienced readers, although some suggested that workflow-level prioritization or routing features might improve perceived utility.

#### Theme 3: Latency and Inconsistent Availability Reduced Usefulness

Multiple respondents noted that AI output was not always available at the time of dictation or arrived too late to be helpful.

#### Theme 4: False-Positive Alerts Increased Second-Guessing and Consultation Burden

Some comments suggested that false-positive AI flags triggered unnecessary consultation or additional cognitive burden.

#### Theme 5: Medicolegal Concerns Influenced Acceptance

Respondents expressed discomfort with the possibility that delayed or discrepant AI outputs could create liability concerns.

#### Theme 6: Respondents Wanted Better Localization, Higher Specificity, and More Training

Suggested improvements included more precise localization, lower false positive rates, better timing, and implementation feedback or training.

#### Aggregated Themes

Aggregated qualitative themes and representative excerpts are summarized in Table 2.

**Table 2:** Qualitative themes from free-text responses. Comments could map to more than one theme; counts reflect the number of comments mentioning each theme at least once (N=65)^a^.

Theme	Description	Count, n (%)	Representative excerpt
False positives or low specificity	Frequent false-positive alerts, commonly attributed to artifacts or calcification; perceived as disruptive and time-consuming	22 (33.8)	“False positives are too frequent...”
Limited utility, discontinue, or low value	Negative overall sentiment, including requests to discontinue the tool or concerns that its value did not justify its burden	13 (20)	“Please remove AI completely.”
Latency or availability	AI^b^ output was sometimes delayed or unavailable at the time of dictation or interpretation	7 (10.8)	“Sometimes AI image made available very late...”
Medicolegal concerns	Concerns about legal vulnerability, accountability, or liability if the AI output appeared after sign-off or conflicted with their interpretation	5 (7.7)	“It could potentially ‘frame’ me.”
False negatives or missed hemorrhages	Concern that the tool could miss subtle hemorrhages, particularly smaller or less conspicuous bleeds	4 (6.2)	“I have seen several false negatives.”
Positive or supportive comments	Explicitly positive feedback or support for continued use despite limitations	4 (6.2)	“The AI ICH tool is great!”
Triage, prioritization, or assignment routing	Suggestions that the tool might be more useful if integrated into prioritization or routing workflows	3 (4.6)	“Positive studies should be assigned...and prioritized.”
Consultation burden or second-guessing	Reports that false-positive AI alerts increased consultation burden or second-guessing	3 (4.6)	“I frequently get consults.”
Training or feedback requests	Requests for additional training, implementation guidance, or feedback on use	2 (3.1)	“I would like additional training.”

^a^Aggregated qualitative findings from optional free-text comments are summarized. Because individual comments could mention more than one concern, counts are not mutually exclusive and reflect the number of comments mentioning each theme at least once.

^b^AI: artificial intelligence.

## Discussion

### Principal Findings

In this survey of radiologists exposed to an FDA-cleared ICH AI detection tool in a national teleradiology environment, 3 findings stood out. First, false positive burden was a central driver of dissatisfaction: fewer than 1 in 5 respondents found false-positive alerts infrequent enough to be acceptable, and one-third (22/65, 33.8%) agreed that time spent reviewing false-positive alerts outweighed the tool’s benefits. Second, perceived workflow benefit was limited: only approximately 1 in 10 respondents reported reduced interpretation time despite a larger minority reporting that the tool might reduce the risk of missing subtle hemorrhages during busy shifts. Third, trust in the AI output was strongly conditional. Respondents were willing to trust the AI much more often when it agreed with their own interpretation than when it conflicted with it.

Taken together, these findings suggest that the practical value of this implementation was constrained less by the abstract idea of AI assistance than by the combined effects of false positive burden, timing, and workflow integration. In other words, the tool appears to have been experienced more as an additional signal to manage than as a consistently efficiency-enhancing aid.

### Relationship to Prior Work

These results are consistent with a growing literature showing that post-deployment experience with clinical AI may differ substantially from laboratory or regulatory performance summaries [2-6]. In radiology and other clinical settings, user acceptance is shaped not only by sensitivity and specificity but also by local disease prevalence, the timing of alerts, the interpretability of outputs, and how often the system changes action in a useful direction [5,6]. Scaringi et al [6] described how real-world deployment can produce an “accuracy paradox” in which acceptable technical performance is experienced as poor because positive alerts carry a high false discovery burden in a low-prevalence setting. That framework is highly relevant in this study, where many respondents perceived the false positive rate as a major practical barrier.

These findings also align with human factors literature on alarm burden and trust in automation. High alert burden and repeated false-positive alerts can result in cognitive overhead, desensitization, and reduced perceived usefulness of decision support systems [7,8]. Meanwhile, trust in automation is often selective rather than global; clinicians may accept AI as supportive when it confirms a preexisting judgment but remain reluctant to defer to it when it conflicts with their own assessment [10,11]. Our survey findings fit that pattern closely. However, importantly, because we measured perceptions rather than observed task performance, our data support cautious description of conditional trust patterns, not stronger causal inferences about AI-driven decision change.

### Interpreting the Self-Reported Behavior Item Cautiously

A survey item about scrutinizing AI-negative results less thoroughly does not establish a behavioral phenomenon in the same way that a controlled multi-reader study could. Accordingly, we interpret this result narrowly. The 6.2% (4/65) agreement rate should be read only as the proportion of respondents who self-reported less thorough scrutiny after a negative AI result on that item. It should not be construed as direct evidence of automation-induced complacency or measurable diagnostic underreading. This distinction is important, particularly because self-report on professionally sensitive behaviors may be influenced by social desirability bias [9,10].

At the same time, the item remains informative as a perception signal. Even a small minority of respondents endorsing reduced scrutiny suggests that how negative AI outputs are presented, timed, and contextualized may matter. Future work using controlled reader studies, audit-based designs, or prospective workflow observation would be better suited to determining whether and when AI output measurably alters vigilance or diagnostic behavior.

### Why False Positive Burden Matters So Much in Teleradiology

The teleradiology environment may amplify the operational importance of false positives. High case volume, rapid turnaround expectations, and distributed communication structures can magnify the cost of reviewing spurious alerts or revisiting already completed interpretations. In such settings, an alert that does not change management may still consume attention, create uncertainty, or generate downstream consultation. The free-text comments in this study point to precisely these burdens: artifact-driven overcalls, delayed output, second-guessing, and extra communication work.

This observation has practical implications for how radiology AI tools should be tuned and evaluated. In some settings, maximizing sensitivity may be appropriate, particularly when AI outputs are used to support prioritization or highlight potentially urgent findings; however, if the resulting false positive burden is too high, this may erode clinician acceptance even when the tool performs as intended at a regulatory or technical level [3,4,6]. Therefore, a workflow-sensitive implementation strategy may matter as much as the operating characteristics of the algorithm itself.

### Implications for Implementation

Several implementation lessons emerge from these data. First, specificity matters. The comments suggest that calcification, streak artifacts, motion, and skull-adjacent findings were recurrent sources of false-positive alerts. Improving discrimination in these common failure modes could meaningfully improve user acceptance. Second, interpretability matters. If discrepant alerts are to influence radiologist behavior in a useful way, users may need clearer localization, confidence communication, or other forms of contextual explanation rather than a generic positive signal [4,9]. Third, timing matters. Decision support that appears after a case has effectively been interpreted is less likely to help and more likely to create noise or ambiguity. Fourth, workflow design matters. Some respondents suggested that the tool’s value might be enhanced by workflow-level prioritization or routing features in addition to its use as an overlay during interpretation.

### Subgroup Findings and Their Interpretation

Neuroradiologists were more likely than non-neuroradiologists to endorse the primary end point that false positive review time outweighed benefits, but this finding should be interpreted cautiously. It was the only subgroup result meeting nominal significance, and no exploratory subgroup differences remained significant after false discovery rate correction. Therefore, the most appropriate interpretation is descriptive and hypothesis generating rather than confirmatory. It is possible that subspecialty training influences tolerance for false positives, perceived incremental value, or expectations of tool performance, but this survey was not designed to establish those mechanisms.

### Strengths and Limitations

A strength of this study is that it captured the views of radiologists exposed to the tool in routine clinical practice rather than in an artificial test setting. The inclusion of both structured survey items and free-text comments helped contextualize quantitative responses and identify concrete implementation barriers.

This study also has several limitations. First, the findings are based on self-reported perceptions rather than objective measures of diagnostic performance, reading time, or patient outcomes. Second, the survey instrument was not formally validated, so items should be interpreted at face value rather than as established scales of latent constructs. Third, dichotomizing Likert responses improved interpretability but reduced response granularity. Fourth, the sample reflects a single organizational implementation and may not generalize to other workflows, prevalence environments, or AI products. Fifth, responses may be affected by selection bias and social desirability bias, particularly for items concerning vigilance or reliance. Finally, subgroup analyses were exploratory, and the study was not powered to make strong claims about differences by neuroradiology training.

### Conclusions

Radiologists using an FDA-cleared ICH AI detection tool in a national teleradiology environment reported substantial false positive burden, limited perceived time savings, and strongly conditional trust in AI output. Self-reported reduced scrutiny after negative AI results was uncommon but endorsed by a minority of respondents. The findings suggest that the real-world value of radiology AI tools depends not only on whether they detect disease but also on whether they do so with sufficient specificity, appropriate timing, and workflow-compatible presentation to earn sustained clinician acceptance.

## Data Availability

All data generated or analyzed during this study are included in this published article.

## Abbreviations

AI: artificial intelligence
CT: computed tomography
DECIDE-AI: Developmental and Exploratory Clinical Investigations of Decision Support Systems Driven by Artificial Intelligence
FDA: Food and Drug Administration
ICH: intracranial hemorrhage

## References

[1] Del Gaizo AJ, Osborne TF, Shahoumian T, Sherrier R (2024). Deep learning to detect intracranial hemorrhage in a national teleradiology program and the impact on interpretation time. Radiol Artif Intell.

[2] Pettet G, West J, Robert D, Khetani A, Kumar S, Golla S, Lavis R (2024). A retrospective audit of an artificial intelligence software for the detection of intracranial haemorrhage used by a teleradiology company in the United Kingdom. BJR Open.

[3] Rohren E, Ahmadzade M, Colella S, Kottler N, Krishnan S, Poff J, Rastogi N, Wiggins W, Yee J, Zuluaga C, Ramis P, Ghasemi-Rad M (2025). Post-deployment monitoring of AI performance in intracranial hemorrhage detection by ChatGPT. Acad Radiol.

[4] Garcia GM, Young P, Dawood L, Elshikh M (2026). Head-to-head comparison of 2 artificial intelligence computer-aided triage solutions for detecting intracranial hemorrhage on noncontrast head CT. AJNR Am J Neuroradiol.

[5] Vasey B, Nagendran M, Campbell B, Clifton DA, Collins GS, Denaxas S, Denniston AK, Faes L, Geerts B, Ibrahim M, Liu X, Mateen BA, Mathur P, McCradden MD, Morgan L, Ordish J, Rogers C, Saria S, Ting DS, Watkinson P, Weber W, Wheatstone P, McCulloch P (2022). Reporting guideline for the early stage clinical evaluation of decision support systems driven by artificial intelligence: DECIDE-AI. BMJ.

[6] Scaringi JA, McTaggart RA, Alvin MD, Atalay M, Bernstein MH, Jayaraman MV, Jindal G, Movson JS, Swenson DW, Baird GL (2025). Implementing an AI algorithm in the clinical setting: a case study for the accuracy paradox. Eur Radiol.

[7] Sendelbach S, Funk M (2013). Alarm fatigue: a patient safety concern. AACN Adv Crit Care.

[8] Ancker JS, Edwards A, Nosal S, Hauser D, Mauer E, Kaushal R (2017). Effects of workload, work complexity, and repeated alerts on alert fatigue in a clinical decision support system. BMC Med Inform Decis Mak.

[9] Bernstein MH, Atalay MK, Dibble EH, Maxwell AW, Karam AR, Agarwal S, Ward RC, Healey TT, Baird GL (2023). Can incorrect artificial intelligence (AI) results impact radiologists, and if so, what can we do about it? A multi-reader pilot study of lung cancer detection with chest radiography. Eur Radiol.

[10] Parasuraman R, Riley V (1997). Humans and automation: use, misuse, disuse, abuse. Hum Factors.

[11] Dzindolet MT, Peterson SA, Pomranky RA, Pierce LG, Beck HP (2003). The role of trust in automation reliance. Int J Hum Comput Stud.

